# Method and validation of synaptosomal preparation for isolation of synaptic membrane proteins from rat brain

**DOI:** 10.1016/j.mex.2014.08.002

**Published:** 2014-08-12

**Authors:** Pradip Kumar Kamat, Anuradha Kalani, Neetu Tyagi

**Affiliations:** Division of Physiology and Biophysics, University of Louisville, School of Medicine, KY 40202, USA

**Keywords:** Brain, Neurons, Synapse protein

## Abstract

The ability to isolate and observe molecular changes in protein composition and function at synapses is important in understanding the disease mechanisms. Because signal transmission is highly regulated by transient phosphorylation of neuronal proteins at the synapse, preservation of this protein modification during synaptosome preparation is essential. Therefore, enriched preparations of synaptic particles called synaptosome are necessary to study synapse function. Because of insufficiency of ample sample for quantitative and qualitative analysis via old method, we applied some modifications that were resultant in high synapse yield. Interestingly, we found that modified methods produced more protein as well as more clear protein band on electrophoresis. Therefore, the modified procedure was better than the older method in effort to isolate more pure synapse protein for improved result outcome.

To advance the method for our study, the following modifications were made to the regularly used protocols:

• The pellet consisting of synaptosomes was cleaned two to three times in HEPES buffer containing proteases inhibitor and centrifuged at 12,000 × *g* for 15 min each. This step is highly essential to remove any contamination of sucrose-HEPES buffer and other organelle's which interfere with protein purification analysis.
• Following this step, the synaptosome pellets were suspended in RIPA buffer (mixed with protease inhibitor and PMSF) along with 0.2% TritonX-100 and further centrifuged at 20,000 × *g*.
• Further, the resulting pellet was discarded and suspended in RIPA buffer (mixed with protease inhibitor and PMSF) only. The sample was immediately used for protein estimation and protein electrophoresis.

## Method details

### Material required

1. Sterile surgical instrument to harvest brain.
2. Syringe pump for cardiac perfusion to make brain free from blood contamination.
3. Chilled normal saline.
4. Labeled sterile petri-dishes kept on ice cube bucket.
5. HEPES buffer.
6. Ultrapure water or autoclaved water.
7. Autoclaved or sterile Eppendorf tube.
8. Centrifuge machine.
9. Protease inhibitor cocktail, RIPA buffer and PMSF.
10. Sodium phosphate buffer for washing of brain sample.
11. Ether anesthesia used to make animal anesthetized.

## Reagent preparation

### Normal saline

Normal saline was prepared by dissolving 0.9% of sodium chloride (NaCl), i.e. 9 g of NaCl in 100 ml of distilled water.

### Sodium phosphate buffer

To prepare the 0.1 M sodium phosphate buffer, 0.2 M of NaOH was added to 250 ml of 0.02 M NaH_2_PO_4_·2H_2_O to adjust pH to 8.0 and volume made up to 500 ml with distilled water (DW). To prepare the sodium phosphate buffer of 0.03 M, pH 7.0. 150 ml of 0.1 M sodium phosphate buffer (pH 8.0) was taken and pH was adjusted to 7.0 and volume made up to 500 ml with DW.

### HEPES buffers

HEPES-buffer solution: 145 mM NaCl (8.41 g), 5 mM KCl (37.2 mg), 2 mM CaCl_2_ (22.1 mg), 1 mM MgCl_2_ (9.51 mg), 5 mM glucose (9.0 mg), 5 mM HEPES (1.19 g), pH 7.4 dissolved in DW, volume made up to 1000 ml.

### Sucrose gradient preparation

1.3 M sucrose: 455.83 g of sucrose in 1000 ml HEPES buffer
0.3 M sucrose: 109.53 g of sucrose in 1000 ml HEPES buffer
0.8 M sucrose: Mixed the solution 1.3 M sucrose and 0.8 M sucrose in 1:1 ratio.

### Radio immune assay precipitation buffer (RIPA)

The RIPA buffer (mixed with phenyl methyl sulphonyl fluoride (10 Mm) and proteases inhibitor cocktail) was used for protein sample preparation for western blotting (Cat No. #p8340; Sigma Aldrich, USA).

### Synaptosomal preparation

Synaptosomes are commonly used to study synaptic function because they contain functional ion channels, receptors, enzymes and proteins, as well as the intact molecular machinery for the release, uptake and storage of neurotransmitters. The method was followed as described by McGovern et al. [1] with some modification to advance the technique.

### Regular procedure

**Fig. 1:**
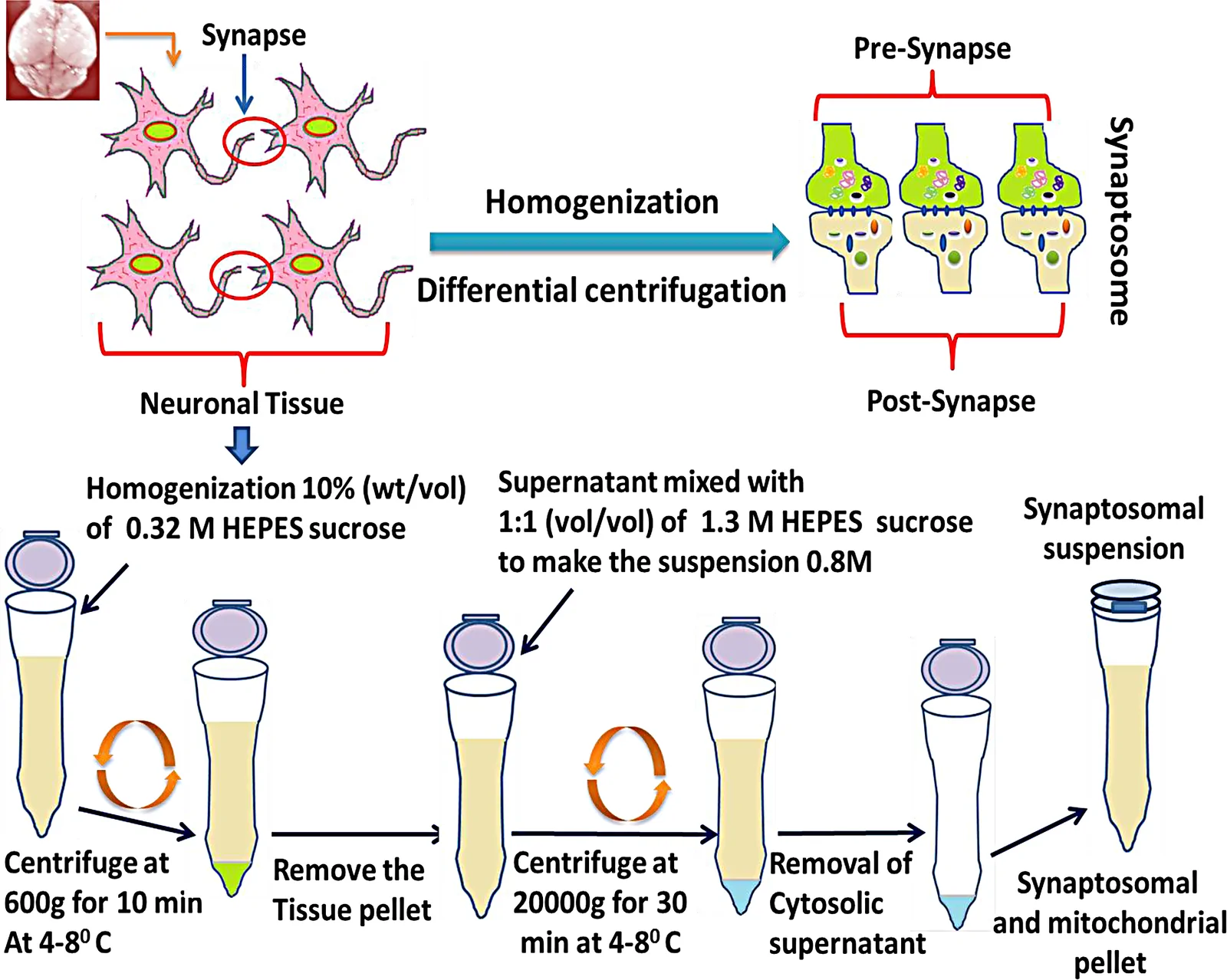
This cartoon illustrates regular methods of synapse preparation. Method was represented by the step-by-step synapse protein preparation from brain and isolation of synapse protein by differential centrifugation. Isolated brain tissues were used for the synapse preparation. Moreover, we described the detail of procedure in methods section.

1. The rats were sacrificed under ether anesthesia and blood was drained by intra cardiac perfusion with chilled normal saline.
2. The brain was removed quickly and kept on ice-cold plate immediately.
3. The brain tissue was suspended in 10% (w/v) of 0.32 M sucrose HEPES buffer in a polytron homogenizer along with protease inhibitor.
4. Suspended tissue was homogenized with Dounce tissue grinder with 10 up-and-down even strokes.
5. Further, homogenate was centrifuged at 4–8 °C for 10 min at 600 × *g* in a 3K300 centrifuge.
6. The supernatant was then diluted 1:1 with 1.3 M sucrose HEPES buffer, to yield a suspension at a final concentration of 0.8 M sucrose.
7. This suspension was further centrifuged at 20,000 × *g* for 30 min at 4 °C. The supernatant was discarded.
8. The pellet consisting of synaptosomes and free (extrasynaptosomal) mitochondria was suspended in RIPA buffer (mixed with protease inhibitor and PMSF). The resulting synaptosomal preparation was used to estimate synapse protein and for other biochemical assay (Fig. 1). During the next day the protein was estimated and used for electrophoresis.

### Modified procedure

This modified procedure contains first two similar steps which are mentioned in the above procedure (Regular Procedure).

1. The rats were sacrificed under ether anesthesia and blood drained out by intra cardiac perfusion with ice-cold normal saline up to 50 ml of volume.
2. The brain was removed quickly, washed with normal saline and kept on ice-cold glass plate immediately.
3. The brain tissue was suspended in 10% (w/v) of 0.32 M sucrose-HEPES buffer in a polytron homogenizer along with protease inhibitor.
4. Suspended tissue was homogenized with Dounce tissue grinder with 10 up-and-down even strokes.
5. Further, homogenate was centrifuged at 4–8 °C for 10 min at 600 × *g* in a 3K300 centrifuge.
6. The supernatant was then diluted 1:1 with 1.3 M HEPES sucrose, to yield a suspension at a final concentration of 0.8 M HEPES sucrose.
7. This suspension was further centrifuged at two to three times at 12,000 × *g* for 15 min at 4 °C. This is a series of washes with HEPES buffer. The supernatant was discarded each time. This step is necessary to remove any contamination of sucrose-HEPES buffer.
8. The pellet consisting of synaptosomes was suspended in RIPA buffer (mixed with protease inhibitor and PMSF) along with 0.2% TritonX-100 and centrifuged at 20,000 × *g* for 30 min. This step is responsible for the disruption of synapse membrane and enriches the synapse protein.
9. The resulting pellet was discarded suspended in RIPA buffer (mixed with protease inhibitor and PMSF) only. The sample was immediately used for protein estimation and protein electrophoresis (Graphical abstract).

### Protein estimation

The protein was estimated in all the brain samples by the method of Lowry et al. [2]. Bovine serum albumin (BSA) 0.01–0.1 mg/ml was used as standard.

### Sample preparation for western blotting of synapse proteins

**Fig. 2:**
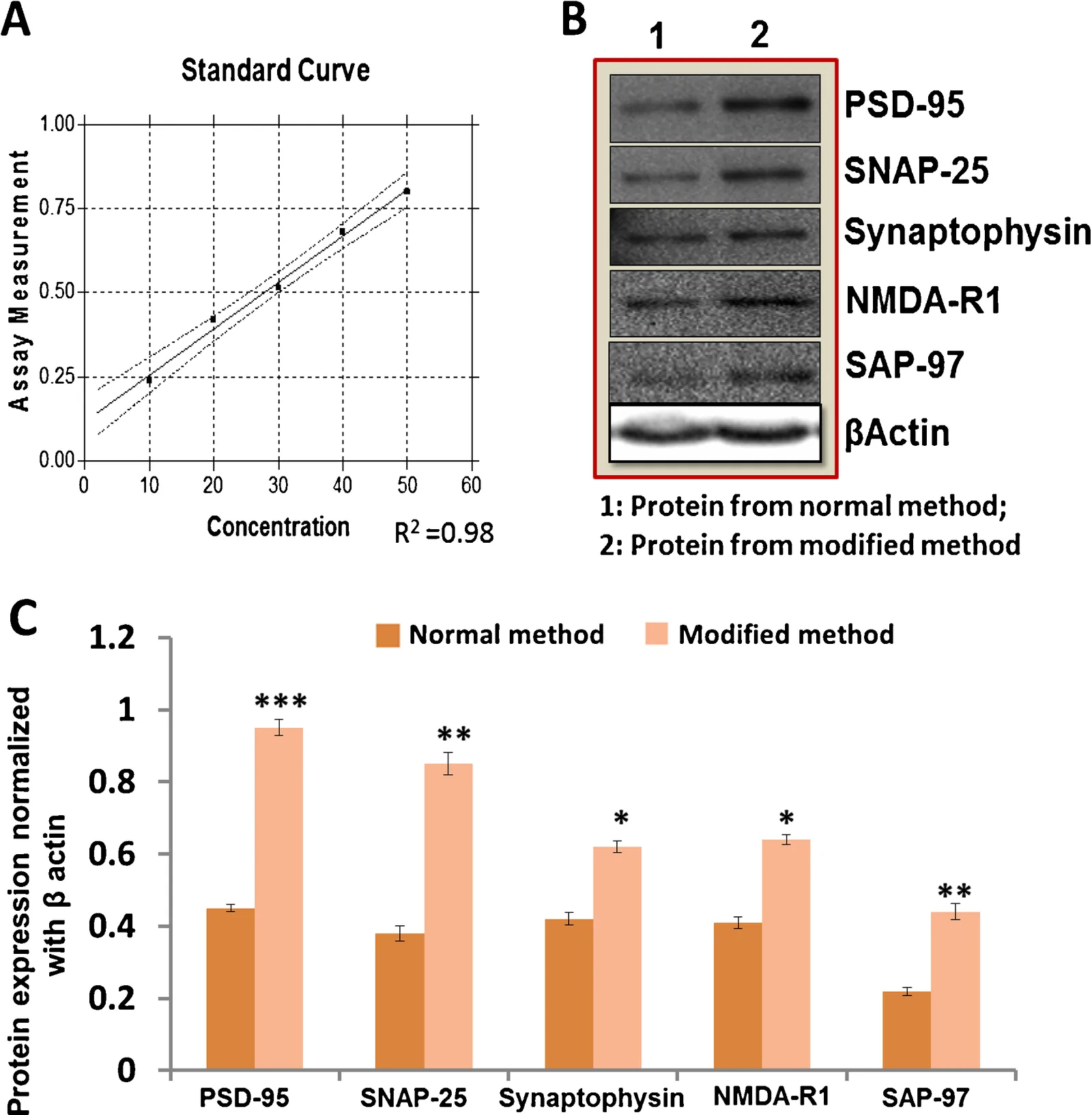
Diagram (A) shows the concentration of the protein which was prepared by bovine serum albumin (BSA) standard curve used for protein estimation. Moreover, we test the protein electrophoresis by old and new method and found that there was difference in intensity of protein bands. Figure (B) depicts the expression of synapse protein by normal procedure as well as modified procedure and figure (C) represents quantitative analysis of protein level. For the quantitative analysis of protein; we used (*n* = 7) sample. Bar graph (C) represents densitometry analysis of PSD-95, SNAP-25, synaptophysin, NMDA-R1 and SAP-97. Data represents mean ± SE from *n* = 7 per group. *P < 0.05, **P < 0.005, ***P < 0.001 vs. normal method.

Equal amount of proteins were loaded for SDS-PAGE for separation, followed by transfer onto a nitrocellulose membrane. The membrane was kept in 1% BSA containing, Tris-buffer saline (TBS), pH 7.6 and then incubated overnight at 4 °C on slow shaking plate. Following the incubation, the membrane was washed with washing buffer (pH 7.6, TBS, 0.1% Tween 20) for 60 min and incubated with synapse protein known primary antibodies against (synaptophysin, SAP-97, SNAP-25, NMDA-R1, PSD-95) and β-actin (1:500 dilution, Santa Cruz Biotechnology, Inc.) for overnight at 40 °C, again washed with TBS-T buffer for 30 min (three washes each of 10 min) and then incubated with secondary antibody conjugated with horseradish peroxidase (1:1000 dilution, Santa Cruz Biotechnology, Inc.) for 2 h at room temperature. This was followed by washing with TBS-T for 30 min (three washes each of 10 min). Then after that those membranes were reacted with ECL reagents (Pierce Biotechnology, Inc.). Relative optical density of protein bands was analyzed using gel documentation system. Sample loading for synapse proteins were normalized with relative optical density of the β-actin band (Fig. 2B). Further quantitative analysis of protein band was done by image Lab version.3 software. Quantification of protein blots was represented by bar graph (Fig. 2C).

## Additional information

Synapse is a structure that permits a neuron to communicate electrical or chemical signal to another neuronal cell and are essential to neuronal function. Synapse loss is an early and critical event of neurological disorder such as Alzheimer's disease (AD) [3], Parkinson's disease (PD) and Huntington's disease (HD). Proper synapse function is important for neuronal viability, and their disruption may account for the cell loss in the later phases of the AD. Biochemical analysis has also revealed a similar loss of both pre-synaptic and post-synaptic components indeed; synaptic degeneration appears to be an early event in pathogenesis with synapse loss evident in patients with neurological disorder. To study the synapse loss in neurodegenerative disease; investigators have developed the method that allows isolation of intact synaptosomes within a crude synaptosomal preparation by different methods [4], [5], [6]. An enriched fraction of synaptic proteins can be obtained from isolated nerve terminals (synaptosomes) prepared by neuronal tissue homogenization. Synaptosomes consist of the presynaptic terminal, including mitochondria and synaptic vesicles, with the postsynaptic membrane and the postsynaptic density proteins [7]. The modified technique described in this manuscript is also useful to analyze the toxic effect of chemical compounds or drugs on synapse function.
